# Proteinuria changes in kidney disease patients with clinical remission during the COVID-19 pandemic

**DOI:** 10.1371/journal.pone.0250581

**Published:** 2021-04-23

**Authors:** Nobuo Tsuboi, Takaya Sasaki, Naoki Kashihara, Takashi Yokoo

**Affiliations:** 1 Division of Nephrology and Hypertension, The Jikei University School of Medicine, Tokyo, Japan; 2 Division of Nephrology and Hypertension, Kawasaki Medical School, Okayama, Japan; Istituto Di Ricerche Farmacologiche Mario Negri, ITALY

## Abstract

**Backgrounds:**

Data on how lifestyle changes due to the coronavirus disease 2019 (COVID-19) pandemic have influenced the clinical features of kidney disease patients remain scarce.

**Methods:**

This study retrospectively analyzed clinical variables in patients with stage G1–G4 chronic kidney disease (CKD) with complete or incomplete remission of proteinuria, who were managed in a nephrology outpatient clinic of a university hospital in Tokyo. The clinical variables during the COVID-19 pandemic (term 1, June–July 2020) were compared to those one year before the pandemic (term 0, June–July 2019). The urinary protein excretion (UPE) was used as the primary outcome measure.

**Results:**

This study included 325 patients with stage G1–G4 CKD (mean age 58.5 years old, 37.5% female, 80.6% on renin-angiotensin aldosterone system inhibitors [RAASis], 12.0% on maintenance dose immunosuppression therapy) evaluated at term 0. The UPE at terms 0 and 1 was 247 (92–624) and 203 (84–508) mg/day [median (25^th^–75^th^ percentile)], respectively; the value in term 1 was 18% lower than that in term 0 (*p*<0.001), with no marked difference in body weight, blood pressure, protein intake or urinary salt excretion. In multivariable analyses, incomplete remission of proteinuria in term 0 (odds ratio [OR] = 2.70, *p* = <0.001), RAASi use (OR = 2.09, *p* = 0.02) and decreased urinary salt excretion in term 1 vs. term 0 (OR = 1.94, *p* = 0.002) were identified as independent variables associated with reduced UPE in term 1 vs. term 0. No significant interactions between the variables were observed.

**Conclusion:**

In kidney disease patients receiving standard medical care from nephrologists, the UPE after the emergency declaration in relation to the COVID-19 pandemic was lower than before the declaration. The UPE reduction may be associated with reduced dietary salt intake during the pandemic in patients treated with RAASi for insufficient control of proteinuria. Our results support the current proposal to continue therapeutic approaches to these patients, which involve RAASi therapy along with optimizing dietary habits, even while dealing with the COVID-19 pandemic.

## Introduction

Coronavirus disease 2019 (COVID-19) emerged in China in December 2019 and spread worldwide within months [1]. Due to the strong infectivity and high mortality rate, COVID-19 pandemic has become a serious social crisis. In Japan, the COVID-19 pandemic began in late March 2019 and rapidly spread to most areas of the country [2,3]. The Japanese government declared a state of emergency for the pandemic on April 7^th^, 2020. Since then, most people have begun to change their lifestyle and to perform social distancing, regardless of the absence or presence of underlying diseases [3].

In patients with kidney diseases, factors associated with physical activity and diet are known to influence clinical variables, such as urinary protein excretion (UPE) [4,5]. Drastic lifestyle changes in the current social crisis due to the COVID-19 pandemic may have had a significant impact, particularly on kidney disease patients, who were attempting to control their daily habits to maintain remission of proteinuria [6,7]. The primary objective of this study was to investigate the changes in UPE that might occur in kidney disease patients under the medical care of nephrologists during the COVID-19 pandemic. Factors potentially involved in the proteinuria changes during the pandemic were then analyzed.

## Materials and methods

### Definition of terms

**Fig 1:**
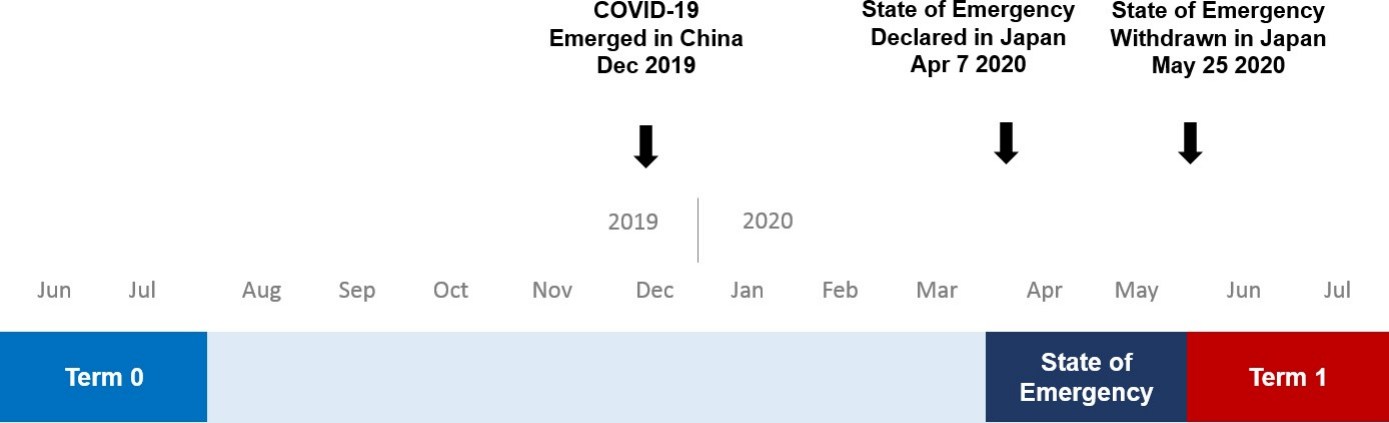
Definitions of terms.

The terms of the study period were defined as follows: term 0, June–July 2019; emergency term, April–May 2020; and term 1, June–July 2020 (**Fig 1**). Data from the emergency term were not included in the analyses, since the number of patient visits in this term were markedly decreased and there was a high probability that the patients’ characteristics or the severity of their disease would introduce biases. In our outpatient clinic, the total number of patient visits before the COVID-19 pandemic was ~3,000 per month. After the declaration of a state of emergency was issued on April 7th, 2020, patient visits in April and May decreased to 56% and 50%, respectively, of the number of visits in the corresponding months in 2019. After the state of emergency was withdrawn, patient visits in June and July 2020 recovered to 86% and 84%, respectively, of the number of visits in the corresponding months of 2019.

### Definitions of clinical variables

Elderly was defined as ≥65 years of age. Obesity was defined as a body mass index (BMI) of ≥25 kg/m^2^ [8]. Hypertension was defined as a systolic blood pressure of >140 mmHg and/or a diastolic blood pressure of >90 mmHg, or the use of antihypertensive drugs. Subjects using antihypertensive medications, such as renin-angiotensin-aldosterone system inhibitors (RAASi), for the purpose of renoprotection despite a normal blood pressure were considered to be normotensive. Diabetes was defined as HbA1c ≥6.5% (NGSP) or the use of antidiabetic therapies, including medications and insulin injection. The estimated GFR (eGFR) was calculated using a modified three-variable equation for the GFR of Japanese individuals [9]: eGFR = 194 × age^-0.287^ × sCr^-1.094^ (× 0.739 if female), where sCr is the serum creatinine level. The chronic kidney disease (CKD) stages were defined, according to the eGFR, as follows: G1 ≥90 mL/min/1.73m^2^; G2, 60–89 mL/min/1.73m^2^; G3a, 45–59 mL/min/1.73m^2^; G3b, 30–44 mL/min/1.73m^2^; G4, 15–29 mL/min/1.73m^2^ and G5, <15 mL/min/1.73m^2^. Therapy with RAASi was defined as the use of an angiotensin-converting enzyme inhibitor or angiotensin type-1 receptor blocker. Immunosuppressive therapy was defined as the use of any immunosuppressive agent, including corticosteroids, irrespective of the duration or dose. The urinary sodium excretion (g/day), estimated protein intake (g/day) and UPE (mg/day) were measured using 24-h urine samples. The 24-h creatinine clearance rate (CCr) was calculated using the measured serum creatinine concentration and urine creatinine concentration in 24-h urine samples. Complete remission and incomplete remission of proteinuria were defined as UPE <300 mg/day and 300 mg/day ≤UPE <3500 mg/day, respectively. Nephrotic-range proteinuria was defined as UPE ≥ 3500 mg/day. Reductions in the UPE, mean arterial pressure, CCr, protein intake and urine salt excretion after the start of the COVID-19 pandemic relative to those values before the pandemic were defined when values in term 1 were less than the values in term 0.

### Patient selection

This retrospective cohort study screened adult patients who visited the nephrology outpatient clinic in Jikei University Hospital, Tokyo, Japan from June 1 to July 31 (term 1) in 2020. CKD stage G1–G4 patients with proteinuria remission (UPE <3500 mg/day as evaluated by 24-h urine samples) with a follow-up of at least 3-months interval were enrolled. Patients were excluded if they met any of the following criteria during the study period (June 2019–July 2020): (i) <1 year of follow-up, (ii) the administration of immunosuppressive induction therapy to obtain remission of proteinuria, (iii) cases in which medications for the maintenance of proteinuria remission were changed or modified, (iv) patients who showed nephrotic range proteinuria (UPE ≥3500 mg/day), (v) patients who required inpatient treatment for any reason, and (vi) patients whose data were not sufficient for the analyses. We provided participants with information about the opportunity to opt out and the need for consent was waived by the ethics committee. This study was approved by the ethics review board of the Jikei University School of Medicine [32–256 (10338)] and conducted according to the Declaration of Helsinki. This study was performed in accordance with the STROBE reporting guidelines.

### Statistical analyses

Variables were presented as the mean ± standard deviation (SD) or median (25^th^–75^th^ percentile) for continuous variables and frequencies and proportions (%) for categorical variables. Corresponding repeated comparisons for each clinical parameter were performed between terms 0 vs. term 1 as appropriate. Wilcoxon’s signed-rank test and McNemar’s test were used to compare changes in the UPE and the rate of patients with complete remission of proteinuria between terms 0 and 1. Univariable and multivariable (backward selection) logistic regression analyses were used to examine the variables associated with a reduced UPE in term 1 compared with term 0. As a sensitivity analysis, univariable and multivariable logistic regression analyses were performed using different definitions of UPE reduction as the dependent variable (>0% decrease or ≥10% decrease in UPE in term 1 relative to term 0). To further examine the interactions among the variables significantly associated with the UPE reduction, multivariable logistic regression analyses were performed by integrating interaction terms as the explanatory variables in the relevant statistical model. All reported *p* values were two-sided. *P* values of <0.05 were considered to indicate statistical significance. All statistical analyses were performed using the SPSS software package (ver. 27.0; IBM, Armonk, NY, USA).

## Results

**Fig 2:**
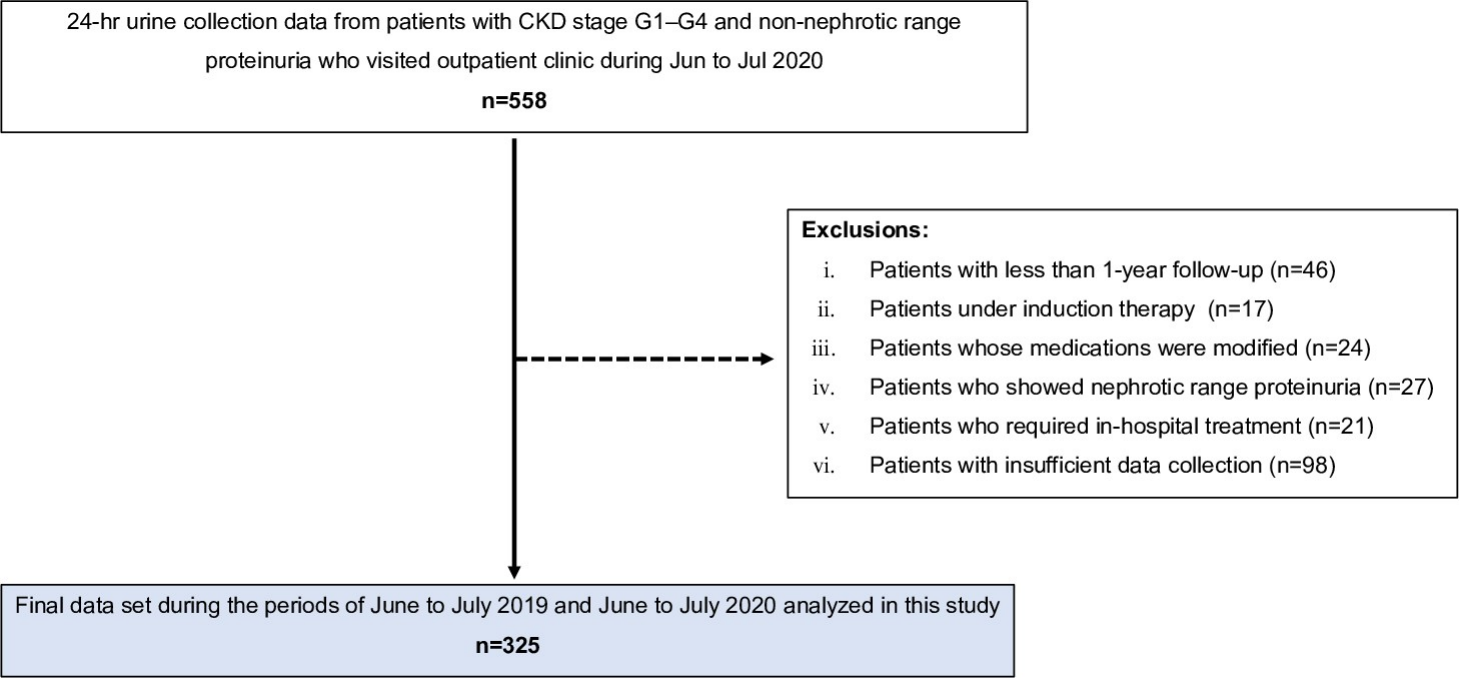
The process of patient selection.

**Fig 2** shows the patient selection process. Among the 5,297 patients who visited our hospital during the inclusion period, we identified 558 patients who fulfilled the inclusion criteria of CKD stage G1–G4 and UPE <3500 mg/day and collected their clinical, serum and 24-hour urine sample data. According to the exclusion criteria, a total of 325 patients were finally selected. All of the patients were Japanese. The clinical characteristics in term 0 are shown in **Table 1**. Overall, the median medical treatment period of the patients at our hospital was 10.8 years, the mean age was 58.5 years old, and 122 (35.7%) of the patients were female. The distribution of the CKD stages was as follows: stage G1, 3.7%; stage G2, 32.0%; stage G3a, 31.4%; stage G3b, 20.0% and stage G4, 12.9%. Renin-angiotensin aldosterone system inhibitors (RAASis) were administered to 262 (80.6%) patients, and maintenance doses of corticosteroids and/or immunosuppressants were administered to 39 (12.0%). One hundred and seventy-six patients (54.2%) had achieved complete remission of proteinuria (UPE <300 mg/day). A histopathological evaluation of a kidney biopsy specimen was performed in 254 (78.2%) patients. IgA nephropathy (n = 140 [43.1%]) was the most common histological diagnosis.

**Table 1:** Clinical variables and histopathological diagnosis of patients enrolled in this study (n = 325).

Valuables	Median (25^th^- 75^th^ percentile), Mean ± SD or n (%)
**Clinical valuables**	
Medical treatment periods, years	10.8 (6.8–17.7)
Age, years	58.5±13.5
Age ≥ 65 years, n (%)	116 (35.7)
Female sex, n (%)	122 (37.5)
BMI ≥ 25 kg/m^2^, n (%)	90 (27.7)
Hypertension, n (%)	136 (41.8)
Diabetes, n (%)	36 (11.1)
CKD stage G1, n (%)	12 (3.7)
CKD stage G2, n (%)	104 (32.0)
CKD stage G3a, n (%)	102 (31.4)
CKD stage G3b, n (%)	65 (20.0)
CKD stage G4, n (%)	42 (12.9)
Patients treated with maintenance doses of corticosteroids or immunosuppressants, n (%)	39 (12.0)
Patients treated with RAAS inhibitors, n (%)	262 (80.6)
Patients with complete remission, n (%)	176 (54.2)
Patients with incomplete remission, n (%)	149 (45.8)
**Histopathological diagnosis**	
**Biopsy-proven, n (%)**	254 (78.2)
IgA nephropathy, n (%)	140 (43.1)
Membranous nephropathy, n (%)	27 (8.3)
Focal segmental glomerulosclerosis, n (%)	18 (5.5)
Minimal change disease, n (%)	15 (4.6)
IgA vasculitis, n (%)	8 (2.5)
Lupus nephritis, n (%)	7 (2.2)
Membranous proliferative glomerulonephritis, n (%)	6 (1.8)
Others or unknown etiology, n (%)	33 (10.2)
**Biopsy-unproven, n (%)**	71 (21.8)

BMI, body mass index; CKD, chronic kidney disease; RAAS, renin-angiotensin aldosterone system; lgA, immunoglobulin A.

**Fig 3:**
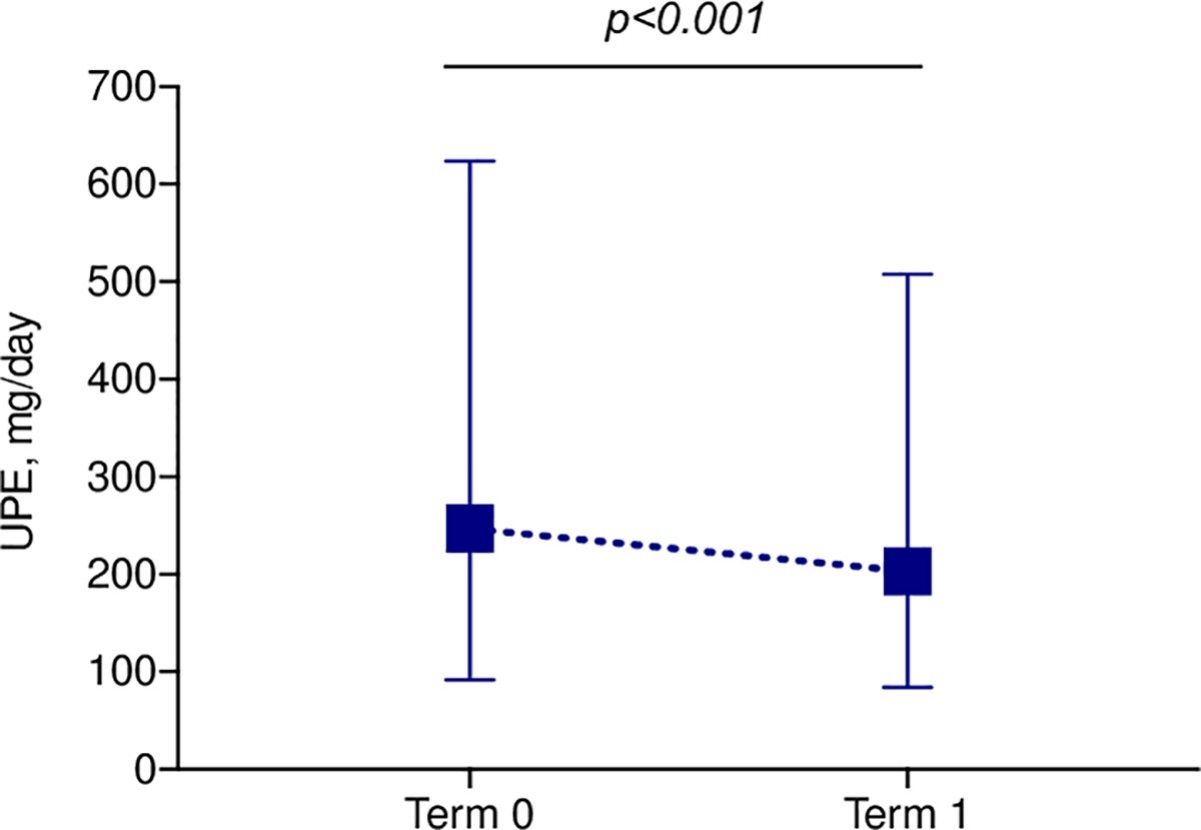
Changes in UPE between terms 0 and 1. The dot and error bars indicate the median and interquartile range.

Comparisons of clinical factors between terms 0 and 1 are shown in **Table 2**. The body weight, blood pressure, protein intake and urinary salt excretion did not differ markedly between the terms. Relative to term 0, the serum creatinine levels increased, urinary creatinine excretion decreased, and the CCr decreased in term 1. The UPE was decreased in 198 patients (60.9%) and unchanged or increased in 127 patients (39.1%) in term 1 relative to term 0. Overall, the UPE in terms 0 and 1 was 247 (92–624) and 203 (84–508) mg/day [median (25^th^–75^th^ percentile)], respectively; the value in term 1 was 18% lower than that in term 0 (*p*<0.001) (**Fig 3**). Complete remission of proteinuria was found in 176 patients (54.2%) in term 0 and in 197 patients (60.6%) in term 1, demonstrating a statistically significant difference (*p* = 0.002).

**Table 2:** Comparison of clinical characteristics among terms (n = 325).

Variables	Term 0 un 2019 –July 2019	Term 1 Jun 2020 –Jul 2020	p-value
Body weight, kg	63.7±13.6	64.0±14.0	0.11
Systolic blood pressure, mmHg	117±13	118±13	0.06
Diastolic blood pressure. mmHg	71±9	70±9	0.74
Serum creatinine, mg/dL	1.17±0.49	1.22±0.54	<0.001
Urinary creatinine excretion, g/day	1.23±0.38	1.22±0.55	0.58
24-hour creatinine clearance rate, ml/min/1.73 m^2^	83.7±33.6	79.0±32.2	<0.001
Protein intake, g/day	62.3±18.6	60.8±18.5	0.10
Urinary salt excretion, g/day	8.4±3.5	8.2±3.6	0.21
Patients with complete remission, n (%)	176 (54.2)	197 (60.6)	0.002

Protein intake and urinary salt excretion were estimated using following equations. Protein intake = [urinary urea nitrogen (mg/dL)/100 × urine volume (L) + (0.031 x body weight] × 6.25 + urinary protein excretion, Urinary salt excretion = urinary sodium (mEq/L) × urine volume (L)/17. Corresponding repeated comparisons were performed for each clinical parameter among the terms 0 vs. 1.

**Fig 4:**
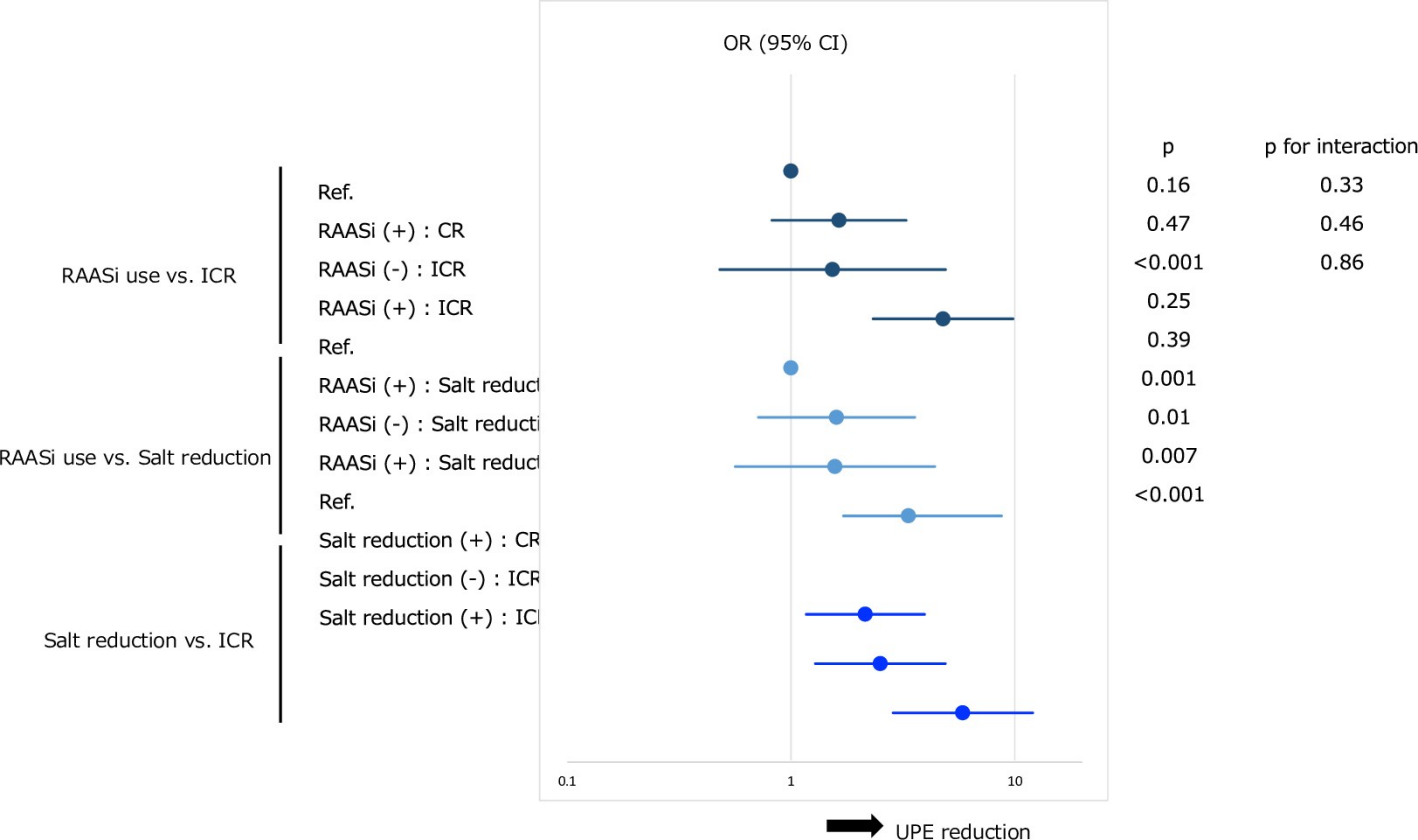
Forest plot of the adjusted ORs with 95% CIs for interaction among clinical variables independently associated with a reduction in UPE in term 1 relative to term 0. Three clinical variables independently associated with reduced UPE in term 1 in comparison to term 0 (RAASi use at term 0, ICR at term 0, and salt reduction in term 1 vs. term 0) were further subjected to multivariable logistic analyses for the interactions. Three combinations of each variable—RAASi use vs. ICR, RAASi use vs. salt reduction, and salt reduction vs. ICR—were analyzed. Patients without either of the variables were used as reference. Log-transformed values for adjusted ORs with 95% CIs, *p* values, and *p* for interactions are presented. Ref., reference; CR, complete remission of proteinuria; ICR, incomplete remission of proteinuria; UPE, urinary protein excretion; RAASi, renin-angiotensin aldosterone system inhibitors; OR, odds ratio; CI, confidence interval.

**Table 3** shows the results of univariable and multivariable logistic analyses of the variables associated with a reduced UPE in term 1 relative to term 0. Among the variables included, incomplete remission of proteinuria in term 0 (odds ratio [OR] = 2.70, *p =* <0.001), RAASi use (OR = 2.09, *p =* 0.02), and decreased urinary salt excretion in term 1 vs. 0 (OR = 1.94, *p =* 0009) were identified as significant variables by backward selection. The same analyses were performed using different definition in the UPE decrease in term 1 relative to term 0 (≥10% decrease in UPE), and similar results were obtained, except that female gender was additionally identified as an independent variable associated with UPE reduction (**S1 Table** in **Supporting Information**). As shown in **Fig 4**, the adjusted ORs for the UPE reduction in term 1 relative to term 0 were significantly higher in patients with any combination of the variables (i.e. RAASi use vs. ICR, RAASi use vs. salt reduction, and salt reduction vs. ICR) than those with either of the variables. No significant interactions between the variables were observed. The detailed data of the **Fig 4** was presented in **S2 Table** in **Supporting Information**.

**Table 3:** Univariable and multivariable logistic regression analyses of variables associated with UPE decrease in term 1 relative to term 0 (n = 325).

Variables	Univariable			Multivariable (backward selection)		
	OR	95% CI	p-value	OR	95% CI	p-value
**Characteristics at term 0**						
Age < 65 years, yes	1.54	0.97–2.46	0.07	-	-	-
Female gender, yes	1.37	0.86–2.19	0.18	-	-	-
BMI ≥ 25 kg/m^2^, yes	1.73	1.03–2.91	0.04	-	-	-
Hypertension, yes	0.86	0.55–1.35	0.86	Not selected		
Diabetes, yes	1.15	0.56–2.37	0.70	Not selected		
eGFR < 60ml/min/1.73m^2^, yes	1.45	0.91–2.31	0.11	-	-	-
Incomplete remission (0.3 ≤ UPE < 3.5g/day), yes	2.82	1.76–4.51	<0.001	2.70	1.65–4.43	<0.001
**Therapies during term 0 to term 1**						
Maintenance doses corticosteroid and/or immunosuppressants, yes	1.17	0.58–2.34	0.67	Not selected		
RAAS inhibitors, yes	2.51	1.43–4.39	0.001	2.09	1.14–3.80	0.02
**Changes in term 1 relative to term 0**						
Decrease in body weight, yes	0.86	0.52–1.43	0.57	Not selected		
Decrease in mean arterial pressure, yes	1.94	1.22–3.06	0.12	-	-	-
Decrease in creatinine clearance, yes	1.43	0.91–2.25	0.005	-	-	-
Decrease in protein intake, yes	2.04	1.30–3.20	0.002	-	-	-
Decrease in urinary salt excretion, yes	2.22	1.41–3.50	0.001	1.94	1.18–3.18	0.009

Variables with p-value < 0.2 in univariable analyses were used for backward selection of multivariable analyses.

BMI, body mass index; CI, confidence interval; OR, odds ratio; RAAS, renin-angiotensin aldosterone system.

## Discussion

This study retrospectively analyzed the changes in clinical variables before and after the emergency declaration for the COVID-19 pandemic in patients with stage G1–G4 CKD with proteinuria remission who were treated in a nephrology outpatient clinic of a university hospital located in Tokyo. Our results showed that the UPE after the emergency declaration for COVID-19 were 18% lower than those evaluated one year before the declaration in these patients, with no marked differences noted in the body weight, blood pressure or dietary contents of protein or salt. This study highlighted, for the first time, the UPE changes in kidney disease patients during the current social crisis due to the COVID-19 pandemic.

This study aimed to investigate the changes in UPE that might occur in kidney disease patients under the medical care of nephrologists during the COVID-19 pandemic. The mechanisms and factors involved in the proteinuria excretions are quite diverse [10]. We therefore applied a strict criteria for the patient selection to obtain enough clinical information. All of the patients included in this study maintained complete or incomplete remission of proteinuria (UPE <3500 mg/day) evaluated at least 3-months interval during the study period (June 2019–July 2020). In addition, patients were restricted to those with CKD stage G1–G4 as patients with advanced kidney dysfunction could often show uncontrolled proteinuria levels independent of changes in lifestyle factors. This study also excluded patients who were under immunosuppressive induction therapy for proteinuria remission or who required changes of drugs for the maintenance of proteinuria remission, to avoid the influence of factors specific to kidney disease activities. Thus, a result showing a reduction in UPE levels after the emergency declaration is likely to be attributed to changes in lifestyle factors during the COVID-19 pandemic.

The COVID-19 pandemic emerged in Japan during winter, in March 2020. Seasonal changes in environmental or patient lifestyle factors may influence the clinical features of many disease conditions, including proteinuria levels in kidney diseases [11]. Our results showed that the UPE after the declaration was significantly lower than that in the same term (June–July) one year before the declaration, suggesting a significant reduction in the UPE beyond the effects of seasonality. A multivariable model suggested that incomplete remission of proteinuria, RAASi use, and decreased urinary salt excretion during the pandemic may be characteristics of patients who showed a reduced UPE during the pandemic relative to that before the pandemic. Changes in body weight, blood pressure, renal function and protein intake were not associated with proteinuria reduction. Same analyses using a different definition of UPE decrease as the explanatory variable (≥10% decrease in UPE levels) confirmed similar results (**S1 Table** in **Supporting Information**). One possible interpretation of this finding is that the current lifestyle during the COVID-19 pandemic may better benefit patients showing these clinical characteristics, i.e., incomplete remission of proteinuria, RAASi use, and decreased urinary salt excretion during the pandemic, with respect to UPE reduction.

The adjusted ORs for the UPE reduction were significantly higher in patients with any combination of the variables than those with either of the variables but did not show any significant interactions, suggesting the additive effects of the variables. These results are consistent with the findings showing that RAASi therapy for reducing overt proteinuria in CKD patients is more effective under salt restricted diet [12–14]. The UPE reduction may be therefore associated with reduced dietary salt intake during the pandemic in patients treated with RAASi for insufficient control of proteinuria. On the other hand, it has been reported that increased salt intake is associated with disaster hypertension and the related increase in albuminuria levels [15]. Thus, our present results may support the current proposal to continue therapeutic approaches for hypertension and CKD, which involve RAASi therapy along with optimizing dietary habits, even while dealing with the COVID-19 pandemic [16–20]. Since no patients was proved to have COVID-19 at the time of patient selection, we could not examine the influences of RAAS inhibitors or salt restricted diet to the clinical findings in kidney disease patients affected by SARS-COV-2 infection in this study cohort.

The present study was associated with several limitations. First, the study population was restricted to patients who underwent routine evaluations of 24-hour urine sample and maintained complete or incomplete remission of proteinuria, and who were expected to have a higher awareness of the importance of self-management for disease control. Additionally, patients were selected from those visited in term 1 when the patient visits had recovered to 84–86% relative to the corresponding months in 2019 in our hospital, which may have produced a bias in the patients’ characteristics. Second, this study indirectly indicated the effects of changes in physical activity on the proteinuria remission status, although we could not estimate the changes in physical activities before the emergency declaration or those after the declaration in this study population. Thus, possibility remains that changes in physical activity, such as changing from working in an office to telework, less effort in going to the workplace, and a longer sitting or sleeping time may have additionally influenced to the changes in the UPE levels. Finally, this study was conducted in a single institution and all patients were Japanese. In addition, the majority of the participants had been diagnosed with primary glomerular diseases. To determine the generalizability of our findings, further studies should be performed in larger populations of CKD patients, including patients of different races and countries.

In conclusion, after the emergency declaration by the Japanese government in relation to the COVID-19 pandemic, the UPE in kidney disease patients with proteinuria remission who received standard medical care from nephrologists was lower than that observed before the declaration, thus indicating that lifestyle changes due to the pandemic influenced the proteinuria levels. This study also suggests that certain clinical characteristics, including incomplete remission, RAASi use and a salt-restricted diet, may be associated with the reduction in UPE observed during the COVID-19 pandemic, supporting the current proposal to continue therapeutic approaches to hypertension and kidney disease patients, which involve RAASi therapy along with optimizing dietary habits, even while dealing with the COVID-19 pandemic.

## Supporting information

S1 Table Univariable and multivariable logistic regression analyses of variables associated with ≥10% UPE decrease in term 1 relative to term 0. (DOCX)

S2 Table Adjusted ORs with 95% CIs for interaction among clinical variables independently associated with a reduction in UPE in term 1 relative to term 0. (DOCX)
