# Correction: Proteinuria changes in kidney disease patients with clinical remission during the COVID-19 pandemic

**DOI:** 10.1371/journal.pone.0256255

**Published:** 2021-08-11

**Authors:** Nobuo Tsuboi, Takaya Sasaki, Naoki Kashihara, Takashi Yokoo

Fig 4 is incorrect. The authors have provided a corrected version here.

**Fig 4:**
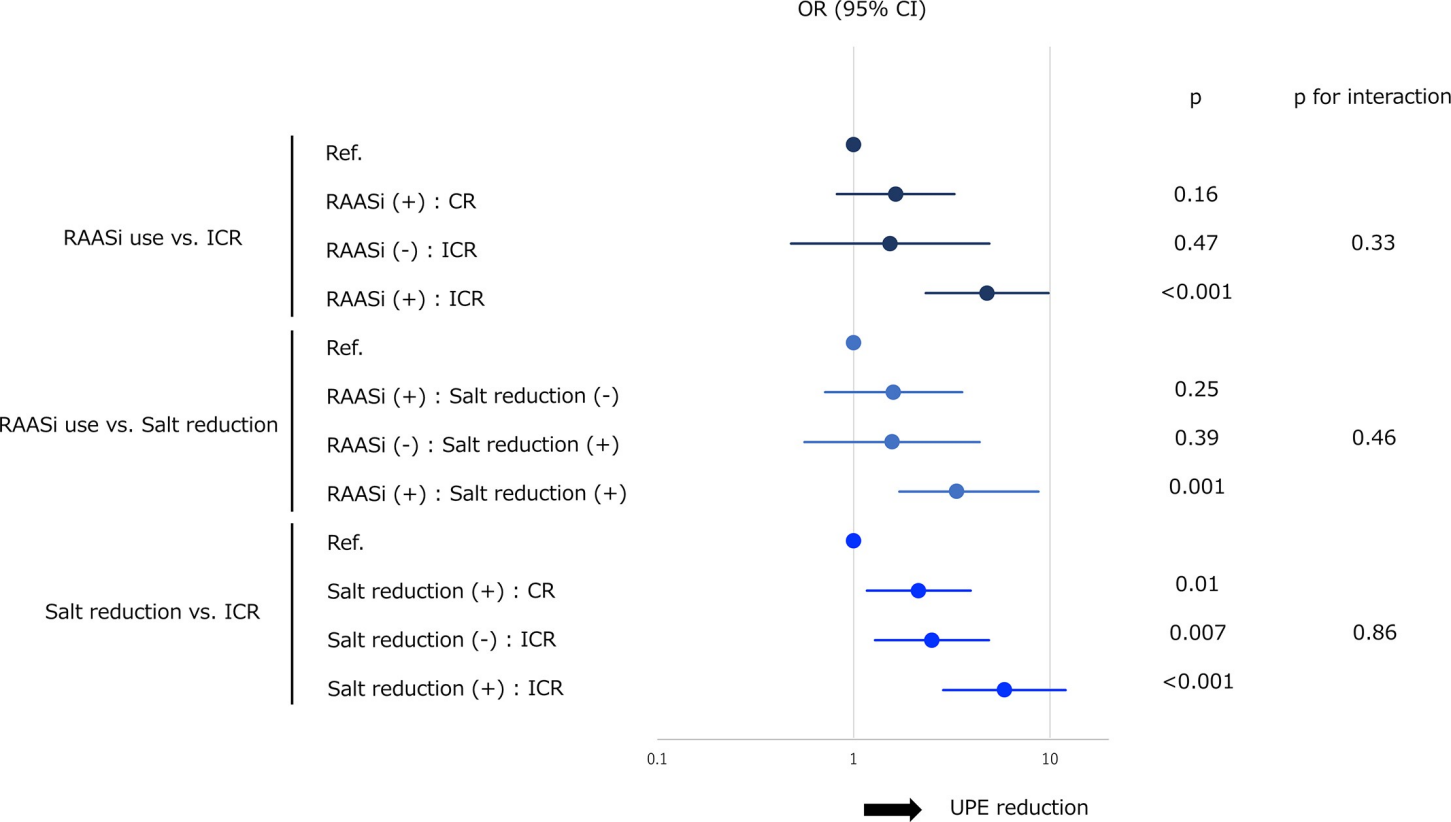
Forest plot of the adjusted ORs with 95% CIs for interaction among clinical variables independently associated with a reduction in UPE in term 1 relative to term 0. Three clinical variables independently associated with reduced UPE in term 1 in comparison to term 0 (RAASi use at term 0, ICR at term 0, and salt reduction in term 1 vs. term 0) were further subjected to multivariable logistic analyses for the interactions. Three combinations of each variable—RAASi use vs. ICR, RAASi use vs. salt reduction, and salt reduction vs. ICR—were analyzed. Patients without either of the variables were used as reference. Log-transformed values for adjusted ORs with 95% CIs, *p* values, and *p* for interactions are presented. Ref., reference; CR, complete remission of proteinuria; ICR, incomplete remission of proteinuria; UPE, urinary protein excretion; RAASi, renin-angiotensin aldosterone system inhibitors; OR, odds ratio; CI, confidence interval.

## References

[1] Tsuboi N, Sasaki T, Kashihara N, Yokoo T (2021) Proteinuria changes in kidney disease patients with clinical remission during the COVID-19 pandemic. PLoS ONE 16(4): e0250581. 10.1371/journal.pone.0250581 33891663 PMC8064597

