# Supplementary material for: Proteinuria changes in kidney disease patients with clinical remission during the COVID-19 pandemic
Source: PLoS One. 2021 Apr 23;16(4):e0250581. doi: 10.1371/journal.pone.0250581 (PMC8064597; doi:10.1371/journal.pone.0250581)
Supplement: S1 Table — (DOCX) [file pone.0250581.s001.docx]

**Table S1. Univariable and multivariable logistic regression analyses of variables associated with >10% UPE decrease in term 1 relative to term 0 (n = 325)**

| **Variables** | **Univariable** | | | **Multivariable (Backward selection)** | | |
| --- | --- | --- | --- | --- | --- | --- |
|  | OR | 95% CI | p-value | OR | 95% CI | p-value |
| **Characteristics at term 0** |  | | |  | | |
| Age < 65 years, yes | 1.62 | 1.02–2.56 | 0.04 | - | - | - |
| Female gender, yes | 1.49 | 0.94–2.35 | 0.08 | 1.69 | 1.04–2.74 | 0.03 |
| BMI > 25 kg/m^2^, yes | 1.47 | 0.90–2.40 | 0.13 | - | - | - |
| Hypertension, yes | 1.07 | 0.69–1.66 | 0.77 | Not selected | | |
| Diabetes, yes | 1.17 | 0.59–2.36 | 0.65 | Not selected | | |
| eGFR < 60ml/min/1.73m^2^, yes | 1.13 | 0.72–1.78 | 0.59 | Not selected | | |
| Incomplete remission (0.3 < UPE < 3.5g/day), yes | 2.30 | 1.47–3.60 | <0.001 | 2.20 | 1.37–3.52 | 0.001 |
| **Therapies during term 0 to term 1** |  | | |  | | |
| Maintenance doses corticosteroid and/or immunosuppressants, yes | 1.38 | 0.70–2.72 | 0.35 | Not selected | | |
| RAAS inhibitors, yes | 2.38 | 1.34–4.22 | 0.003 | 2.06 | 1.13–3.77 | 0.02 |
| **Changes in term 1 relative to term 0** |  | | |  | | |
| Decrease in body weight, yes | 1.16 | 0.71–1.91 | 0.56 | Not selected | | |
| Decrease in mean arterial pressure, yes | 1.42 | 0.92–2.20 | 0.12 | - | - | - |
| Decrease in creatinine clearance, yes | 1.78 | 1.13–2.79 | 0.01 | - | - | - |
| Decrease in protein intake, yes | 1.82 | 1.17–2.83 | 0.008 | - | - | - |
| Decrease in urinary salt excretion, yes | 2.18 | 1.40–3.40 | 0.001 | 2.14 | 1.35–3.40 | 0.001 |

Variables with p-value < 0.2 in univariable analyses were used for backward selection of multivariable analyses.

BMI, body mass index; CI, confidence interval; OR, odds ratio; RAAS, renin-angiotensin aldosterone system
