# Supplementary material for: Proteinuria changes in kidney disease patients with clinical remission during the COVID-19 pandemic
Source: PLoS One. 2021 Apr 23;16(4):e0250581. doi: 10.1371/journal.pone.0250581 (PMC8064597; doi:10.1371/journal.pone.0250581)
Supplement: S2 Table — (DOCX) [file pone.0250581.s002.docx]

**Table S2. Adjusted ORs with 95% CIs for interaction among clinical variables independently associated with a reduction in UPE in term 1 relative to term 0** **(n = 325)**

| **Variables** | **ORs** | **95% CIs** | **p value** | **P for interaction** |
| --- | --- | --- | --- | --- |
| **RAASi use vs. ICR** | | | | |
| RAASi (-): CR | 1.00 (reference) | - | - | 0.33 |
| RAASi (+): CR | 1.64 | 0.82–3.27 | 0.16 |  |
| RAASi (-): ICR | 1.53 | 0.48–4.91 | 0.47 |  |
| RAASi (+): ICR | 4.78 | 2.33–9.30 | <0.001 |  |
| **RAASi use vs. Salt reduction** | | | | |
| RAASi (-) : Salt reduction (-) | 1.00 (reference) | - | - | 0.46 |
| RAASi (+) : Salt reduction (-) | 1.60 | 0.72–3.57 | 0.25 |  |
| RAASi (-) : Salt reduction (+) | 1.57 | 0.56–4.38 | 0.39 |  |
| RAASi (+) : Salt reduction (+) | 3.90 | 1.74–8.73 | 0.001 |  |
| **Salt reduction vs. ICR** | | | | |
| Salt reduction (-) : CR | 1.00 (reference) | - | - | 0.86 |
| Salt reduction (+) : CR | 2.15 | 1.17–3.95 | 0.01 |  |
| Salt reduction (-) : ICR | 2.51 | 1.28–4.89 | 0.007 |  |
| Salt reduction (+) : ICR | 5.86 | 2.86–12.02 | <0.001 |  |

Three clinical variables independently associated with reduced UPE in term 1 in comparison to term 0 (RAASi use at term 0, ICR at term 0, and salt reduction in term 1 vs. term 0) were further subjected to multivariable logistic analyses for the interactions. Three combinations of each variable—RAASi use vs. ICR, RAASi use vs. salt reduction, and salt reduction vs. ICR—were analyzed. Patients without either of the variables were used as reference.

CI, confidence interval; CR; complete remission; ICR, incomplete remission; OR, odds ratio; RAAS, renin-angiotensin aldosterone system
